# Preparation and Microstructural Characterization of a High-Cr White Cast Iron Reinforced with WC Particles

**DOI:** 10.3390/ma13112596

**Published:** 2020-06-06

**Authors:** Aida B. Moreira, Laura M. M. Ribeiro, Pedro Lacerda, Ricardo O. Sousa, Ana M. P. Pinto, Manuel F. Vieira

**Affiliations:** 1 Department of Metallurgical and Materials Engineering, University of Porto, R. Dr. Roberto Frias, 4200-465 Porto, Portugal; up201108098@fe.up.pt (A.B.M.); lribeiro@fe.up.pt (L.M.M.R.); rmsousa@fe.up.pt (R.O.S.); 2 LAETA/INEGI-Institute of Science and Innovation in Mechanical and Industrial Engineering, R. Dr. Roberto Frias, 4200-465 Porto, Portugal; 3 FERESPE, Fundição de Ferro e Aço Lda., Vila Nova de Famalicão, Portugal; pedrolacerda@ferespe.pt; 4 CMEMS-Center for MicroElectroMechanics Systems, Department of Mechanical Engineering, University of Minho, Campus de Azurém, 4800-058 Guimarães, Portugal; anapinto@dem.uminho.pt

**Keywords:** high-chromium white cast iron, ex situ technique, local reinforcement, metal matrix composite, microstructural characterization, tungsten carbide

## Abstract

High-chromium white cast iron (WCI) specimens locally reinforced with WC–metal matrix composites were produced via an ex situ technique: powder mixtures of WC and Fe cold-pressed in a pre-form were inserted in the mold cavity before pouring the base metal. The microstructure of the resulting reinforcement is a matrix of martensite (α’) and austenite (γ) with WC particles evenly distributed and (Fe,W,Cr)_6_C carbides that are formed from the reaction between the molten metal and the inserted pre-form. The (Fe,W,Cr)_6_C precipitation leads to the hypoeutectic solidification of the matrix and the final microstructure consists of martensite, formed from primary austenite during cooling and eutectic constituent with (Fe,Cr)_7_C_3_ and (Fe,W,Cr)_6_C carbides. The presence of a reaction zone with 200 µm of thickness, between the base metal and the composite should guarantee a strong bonding between these two zones.

## 1. Introduction

High-chromium white cast irons (WCIs) are based on the Fe-Cr-C ternary system. The addition of further alloying elements, such as molybdenum, nickel, copper and manganese can significantly affect the microstructure, in what concerns the matrix and type of carbides formed, and consequently the final mechanical properties [1,2,3,4].

The high-Cr WCIs containing 12–30 wt.% Cr are extensively used in severe wear and corrosion applications due to their excellent wear resistance, ability to withstand moderate impact and relatively low production cost. The as-cast microstructure of hypereutectic high-Cr WCIs presents coarse primary M_7_C_3_ carbides and a eutectic structure of γ and fine M_7_C_3_ carbides, which are responsible for the excellent abrasion resistance of these alloys [1,2,4,5,6]. Moreover, further, wear resistance can be achieved by a destabilization heat treatment that causes the transformation of the γ-phase into martensite (α’) and fine carbides of M_7_C_3_ and M_23_C_3_ [4,7,8].

The wear behavior of components made of high-Cr WCIs can be more improved by ceramic reinforcement of the surfaces that will be exposed to wear, maintaining the toughness of the bulk component [9,10,11,12,13,14,15,16,17]. Among the reinforcements that can be applied, WC particles are the most used, combining high hardness (3100–3600 HV), thermal expansion coefficient (4.5–7.1 × 10^−6^ °C^−1^) compatible with the base metal (8–12.5 × 10^−6^ °C^−1^), higher elastic modulus compared with other transition metal carbides and good wettability by molten iron [18,19,20].

The liquid-state process is the most used in the manufacture of metal matrix composites due to lower associated costs and easier manufacturing when compared to solid-state processes, such as powder metallurgy, mechanical alloying, diffusion bonding or roll bonding [21,22,23]. In the liquid-state technique, the molten metal infiltrates through compacted ceramic particles, previously placed in the mold cavity, reacting with it and producing a metal matrix composite. The process can be done by pressureless infiltration (spontaneous or reactive infiltration) [11,12,14,15,24,25,26] or by pressure-driven infiltration (squeeze casting, vacuum pressure casting, centrifugal casting) [13,16,20,27,28,29,30,31,32,33,34,35,36,37,38,39]. The major advantage of the liquid-state process is the possibility of producing products with complex geometry and parts with a surface reinforcement whereas higher wear resistance is needed [21,22,23].

Several investigations have been done on white cast irons parts with local reinforcement of WC particles, focusing on processing aspects [11], mechanical performance [20,24,28,32,33,37], but only a few of them have given a detailed insight in the microstructural characterization [12,15,28]. Kambakas and Tsakiropoulos [15] successfully produced high-Cr WCI reinforced with WC-Co particles. The authors identified the presence of Fe_3_W_3_C, Fe_6_W_6_C and M_7_C_3_ in the final microstructure of the cast parts. Zhang et al. [12] also investigated the reinforcement of high-Cr WCI parts using WC-Co particles, achieving a reinforced zone with 15 mm thickness and a sound bonding interface. Several types of carbides (M_6_C, M_12_C, (Cr, W, Fe)_23_C_6_, WC, W_2_C) were identified within the composite zone. Thus, one might say that the controlling of the process lies on the understanding of the relationship between the phases formed in the composite zone, including the bonding interface and the abrasive wear resistance, which demands a very detailed microstructural analysis. In this sense, the focus of this work is the characterization of the phases formed on a high-Cr WCI reinforced with WC particles by pressureless infiltration technique. For this purpose, powders mixtures of WC and Fe were used, expecting that the Fe powder would act as a flux to improve the infiltration between the molten metal and the WC particles. To date, the use of Fe powder as a flux agent and sodium silicate as a binder, have not been explored in the context of the reinforcement of high-Cr white cast irons, representing one innovative solution for processing these materials.

## 2. Materials and Methods

**Figure 1:**
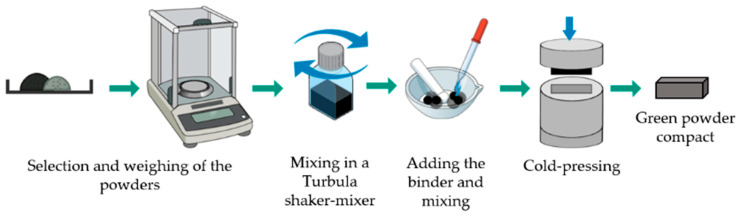
Scheme of the fabrication steps for the green compacts production.

**Figure 2:**
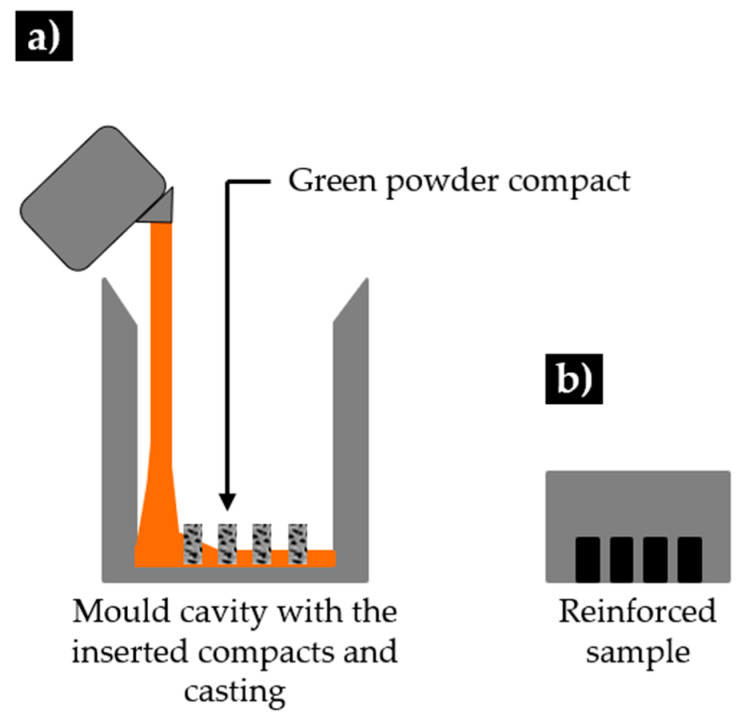
Scheme of the (**a**) mold with the inserted green compacts and (**b**) reinforced specimen (adapted from [40]).

A technique with several steps was applied to produce the reinforced specimens. This technique is schematically presented in Figure 1 and Figure 2. Commercial powders of WC powders (99.0 wt.% purity) and Fe (99.0 wt.% purity), from Alfa Aesar, ThermoFisher (Kandel, Germany) GmbH were used to produce green compacts to be inserted in the mold cavity. First, the morphology and granulometric distribution of the powders were characterized by scanning electron microscopy (SEM), using a FEI QUANTA 400 FEG (FEI Company, Hillsboro, OR, USA) with an energy-dispersive detector (EDS) and dynamic light scattering (DLS, Laser Coulter LS230 granulometer, Beckman Coulter, Inc., Brea, CA, USA). In the second step, the WC and Fe powders were mixed in a volume fraction of 40:60, which, according to the literature, is within the range that it is expected to guarantee the best wear performance. The mixture was homogenized in Turbula shaker-mixer (Willy A. Bachofen AG, Muttenz, Switzerland) for 7 h, and bound with sodium silicate (1.5 mL). Then, the mixture was uniaxially cold-pressed at approximately 70 MPa in a metallic mold to produce green compacts with dimensions of 31 mm × 12 mm × 7 mm. SEM analysis was performed to study the characteristics of both the powder mixtures and the green compacts. The composition of the mixture was selected from data the literature.

In the final step, the green compacts were inserted in the mold cavity, and the high-chromium white cast iron, melted in a medium frequency induction furnace with a capacity of up to 1000 kg, was poured at a temperature of 1460 °C. The nominal chemical composition of the base metal (see Table 1) was analyzed by optical emission spectrometry (MAXx LMM05, Spectro, Germany).

A cross-section of the specimen was cut by wire electrical discharge for visual control of the inner zones of the composite and the bonding between the composite and the base metal. Metallographic samples were prepared and etched with Beraha-Martensite reagent. The microstructure was characterized by optical microscopy (OM) using a Leica DM 4000M with a DFC 420 camera (Leica Microsystems, Wetzlar, Germany) and SEM secondary electron (SE) and backscattered electron (BSE) image. Electron backscatter diffraction (EBSD) analysis has been used to assist with phase identification. The data obtained from EBSD were submitted to a dilation clean-up procedure, using a grain tolerance angle of 15° and the minimum grain size of 10 points, to avoid inaccurate predictions.

A detailed characterization of the phases formed was performed in transmission electron microscopy (TEM) using a JEOL 2100 (JEOL Ltd., Akishima, Tokyo) operated at 200 keV. For that, thin foils were prepared in a dual-beam focused ion beam (FIB) FEI Helios NanoLab 450S (FEI Company, Hillsboro, OR, USA). On TEM, the phases were fully identified through selected area electron diffraction (SAED). X-ray diffraction (XRD, Cu Kα radiation, Bruker D8 Discover), with a scanning range (2θ) of 20° to 100°, was used to complement the characterization of the phases.

## 3. Results and Discussion

### 3.1. Characterization of the Initial Powders

**Figure 3:**
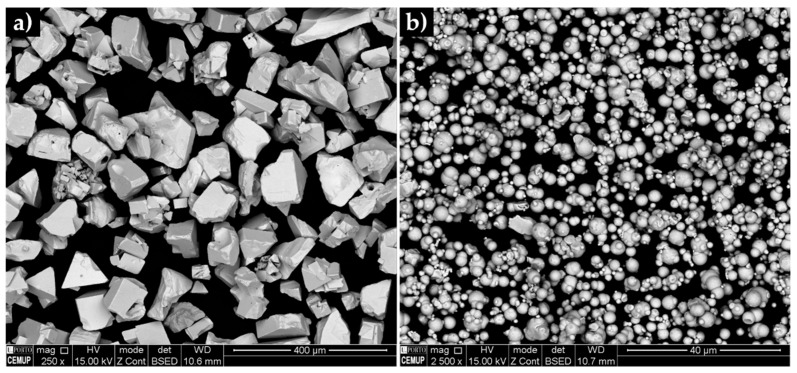
SEM-backscattered electron (BSE) images of the initial powders: (**a**) WC with polyhedral morphology and (**b**) round particles of Fe.

**Figure 4:**
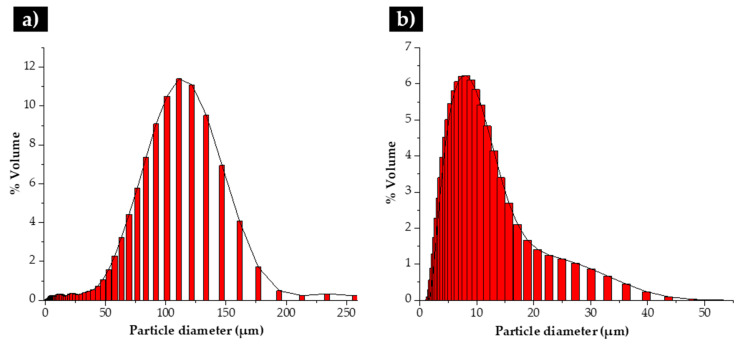
Granulometric distribution of the initial powders: (**a**) WC and (**b**) Fe.

**Figure 5:**
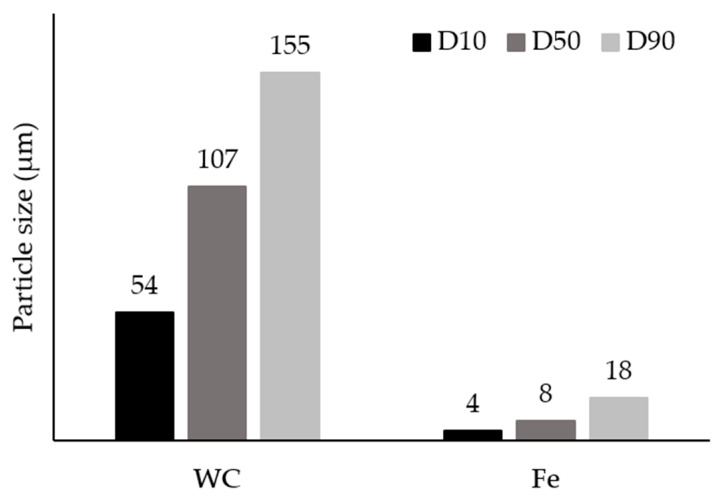
Analysis of the particle size distribution by dynamic light dispersion (DLS), indicating parameters D_10_, D_50_ and D_90_ of the initial powders.

The initial powders were first characterized. Their morphological aspect and size distribution are presented in Figure 3, Figure 4 and Figure 5. From these figures, it is possible to see that WC and Fe powders exhibited different size, shape and granulometric distribution. The WC powders (see Figure 3a) exhibit a roughly polyhedral shape, an average size of 106 µm and a D_50_ of 107 µm (Figure 4a and Figure 5). These results are consistent with the supplier’s specifications (particle size between 53 and 149 µm). The Fe powders exhibit a spherical shape (Figure 3b), an average size of 10 µm and a D_50_ of 8 µm (Figure 4b and Figure 5). To note that, in this case, the results are significantly different from those of the supplier’s specification (particle average size of 74 µm).

### 3.2. Characterization of the Green Compacts

**Figure 6:**
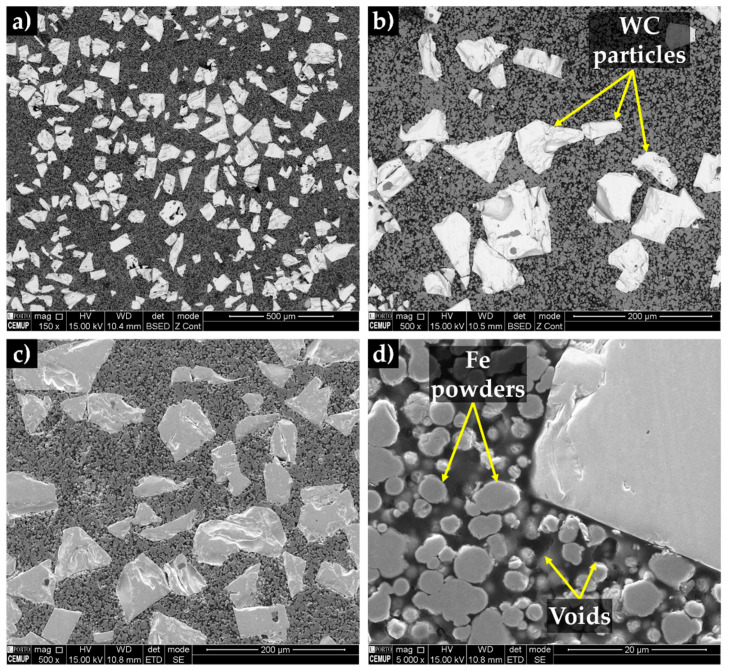
SEM cross-section images of the green compacts: (**a**) low, (**b** and **c**) medium and (**d**) high magnification.

The SEM cross-section images of the compacted powders show white particles of WC, gray particles of Fe (see Figure 6a,b) and dark regions corresponding to the binder phase. Using secondary electron (SE) contrast (see Figure 6c,d), it is possible to observe the presence of voids, which may be useful for the liquid metal infiltration. It can be seen that the binder has spread over the WC particles.

### 3.3. Characterization of the Base Metal

The chemical composition of the base metal, which is classified as an abrasion-resistant cast iron of class III and type A [41] can be found in Table 1.

**Figure 7:**
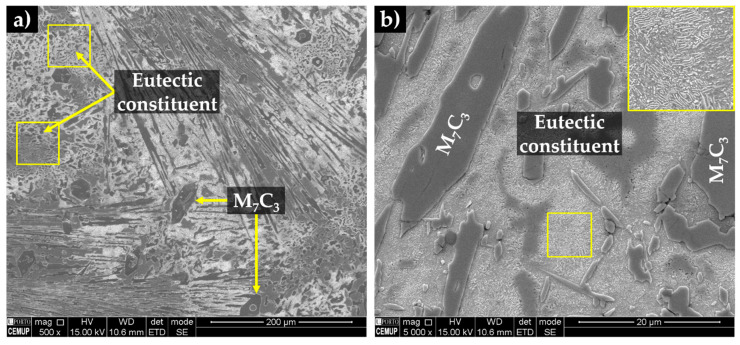
SEM-secondary electron (SE) image of the microstructure of the high-Cr WCI in the as-cast condition (**a**), and at higher magnification (**b**).

**Figure 8:**
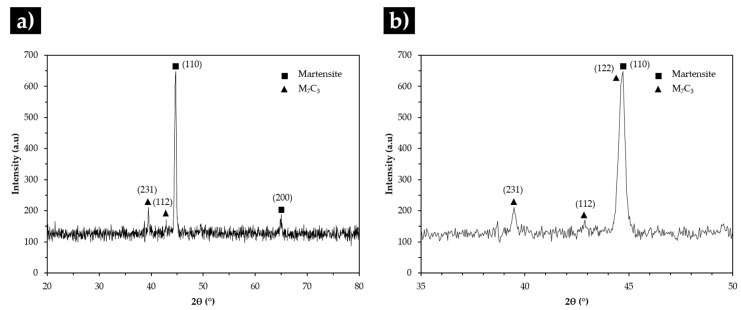
X-ray diffraction (XRD) patterns of the high-Cr WCI in the as-cast condition in the 2θ range of 20°–80° (**a**) and 35°–50° (**b**).

The SEM analysis of the base metal (see Figure 7) revealed large Cr-rich carbides with rod-like structure and eutectic constituent, certainly formed from the eutectic reaction $L\to \gamma +{\left(Fe,Cr\right)}_{7}C_{3}$ [1,2,42]. The plate morphology of the eutectic constituent (observed in Figure 7b) can be explained by the transformation of the austenite on cooling. To clarify this topic, the microstructure was investigated by X-ray diffraction and indexing Kikuchi patterns, using EBSD. The XRD patterns of the base metal presented in Figure 8 provided the evidence for the presence of martensite and Cr-rich carbides with the stoichiometry of M_7_C_3_.

**Figure 9:**
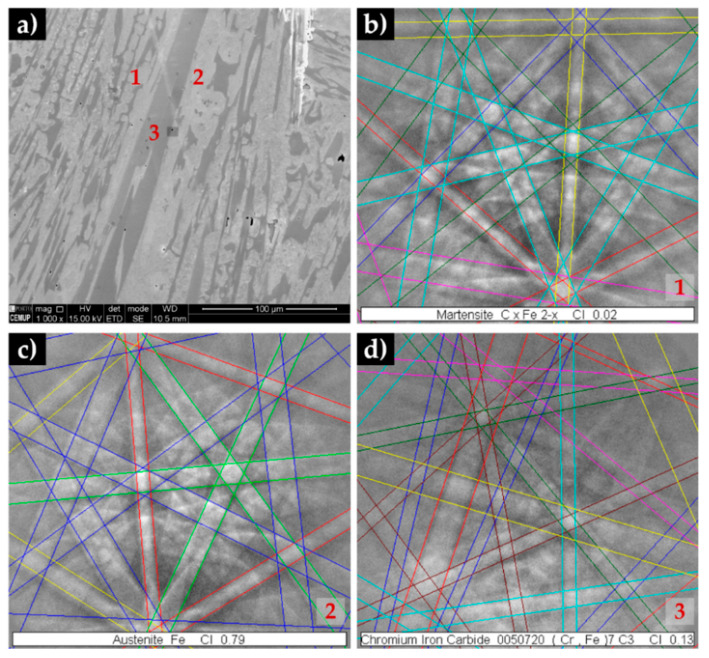
SEM-SE image of the base metal in the as-cast condition (**a**), and indexed EBSD patterns corresponding to the phases that compose the microstructure: α’ (**b**), γ (**c**) and M_7_C_3_ (**d**).

The EBSD results showed in Figure 9, point out the presence of martensite (Figure 9b), Cr-rich M_7_C_3_ carbides (Figure 9d) and also γ phase in some regions, as can be seen in Figure 9c. This phase was not detected in the XRD patterns, indicating that at least its content is less that the detection limit.

**Figure 10:**
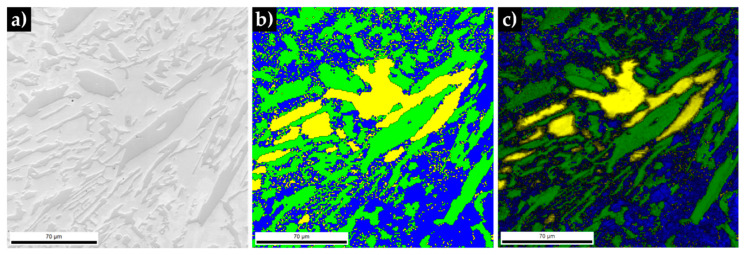
SEM image of the microstructure of the base metal (**a**), phase map: γ in yellow, α’ in blue and M_7_C_3_ in green (**b**) and image quality map (**c**).

In addition to the indexed Kikuchi patterns, the EBSD phase maps of the microstructure were calculated, as shown in Figure 10. The images confirm the previous observations, with the presence of martensite, carbides and austenite being evident. This observation is confirmed by the image quality map (see Figure 10c) that guarantees the accuracy of the diffraction patterns [43].

**Figure 11:**
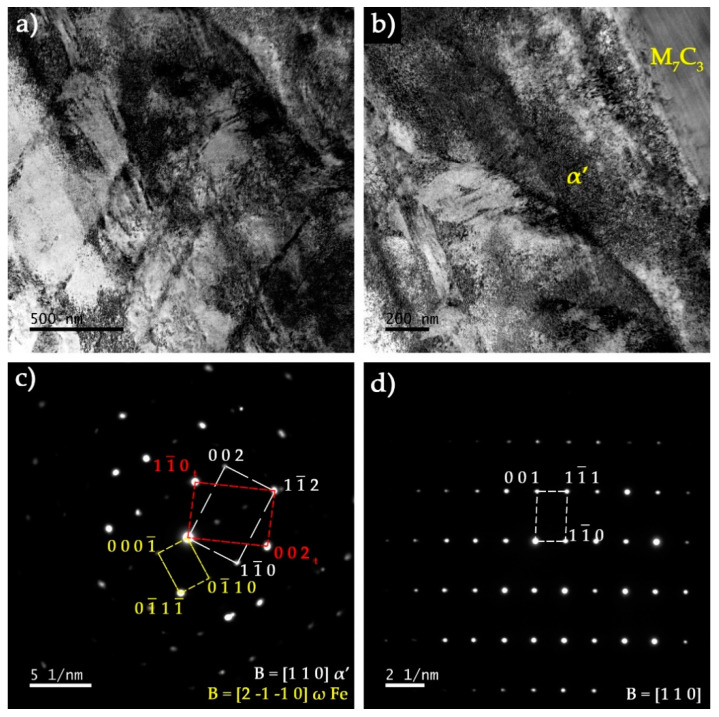
Dark-field TEM image of the base metal showing (**a**) α’ phase revealing twin contrast and (**b**) interface between α’ and a Cr-rich M_7_C_3_ carbide. The identification of phases was concluded by selected area electron diffraction (SAED) analysis with (**c**) [110] zone axis of α’ and (**d**) [110] zone axis of M_7_C_3_.

TEM analysis of thin foils prepared from base metal confirmed the presence of martensite (α’). Figure 11a shows plate martensite (α’) with distinct contrasts that can be attributed to the high density of twins. A particular region showing an interface between α’ and a particle of M_7_C_3_ is presented in Figure 11b. The SAED pattern taken from the particle in shown in Figure 11d. In Figure 11c another interesting aspect can be observed, which is the twin-double-diffraction effect detected in the [110]_α’_ SAED pattern, revealing a BCC {112} <111>-type twins. In this region, extra diffraction spots at 1/3 (−112) and 2/3 (−112) positions, indicated the presence of ω-Fe, which is a metastable hexagonal nano-phase (see yellow spots). The occurrence of ω-Fe phase has been reported to be associated with twinned martensite [44,45,46,47].

### 3.4. Characterization of the Reinforced Specimens

**Figure 12:**
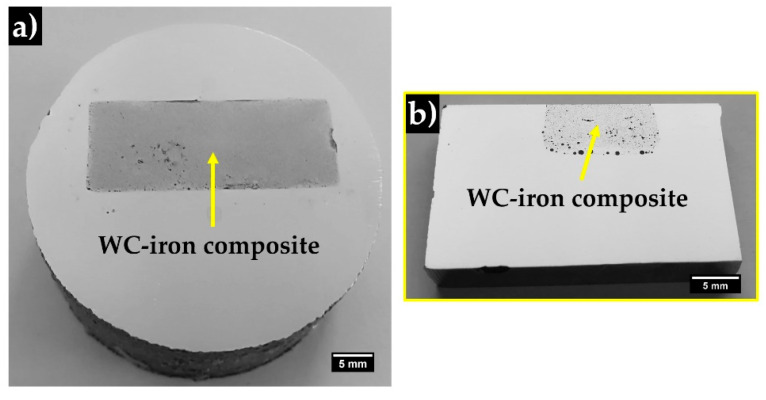
The reinforced cast specimen (**a**) and a cross-section (**b**) to highlight the depth of the composite and the interface zone.

The reinforced cast specimen and a cross-section are presented in Figure 12. In the polished surface, it is possible to distinguish two zones, which are the composite (gray zone) and the high-Cr WCI (light-gray zone). The composite zone exhibits a length and a width of around 31 mm and 12 mm, respectively, and a nearly uniform depth (5 mm), which are close to the green compact dimensions.

**Figure 13:**
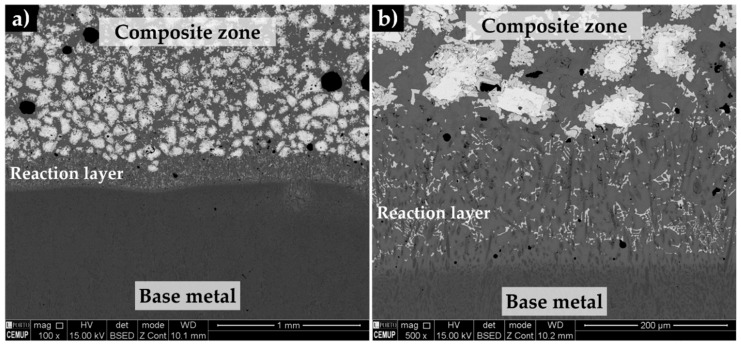
SEM-BSE images of the microstructure of the reinforced specimen showing the base metal and composite zone (**a**) and evidencing the reaction layer (**b**).

The microstructure of the composite and the interface bonding of the composite and the base metal is shown in Figure 13. The bonding interface is free of any discontinuities, such as voids and porosities, suggesting that a good infiltration of the molten metal into the green compact was obtained. Furthermore, the reaction layer between the composite and the base metal is around 200 µm, as could be seen in Figure 13b, suggesting a strong bonding between the composite and the base metal.

**Figure 14:**
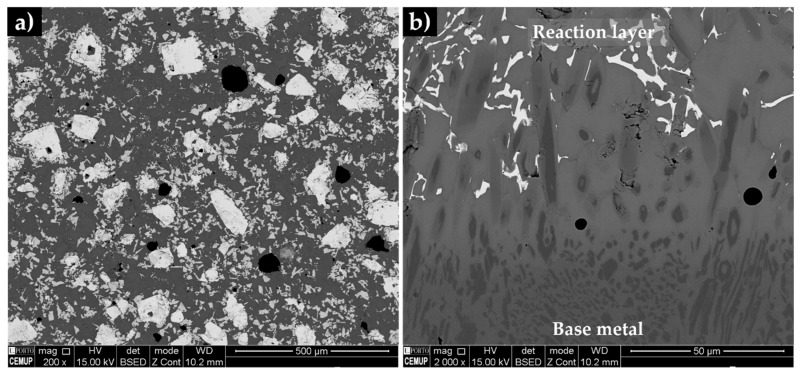
SEM-BSE image of the microstructure of the composite zone, showing a uniform distribution of the carbide particles (**a**) and the reaction layer, evidencing a bonding interface free of discontinuities (**b**).

**Figure 15:**
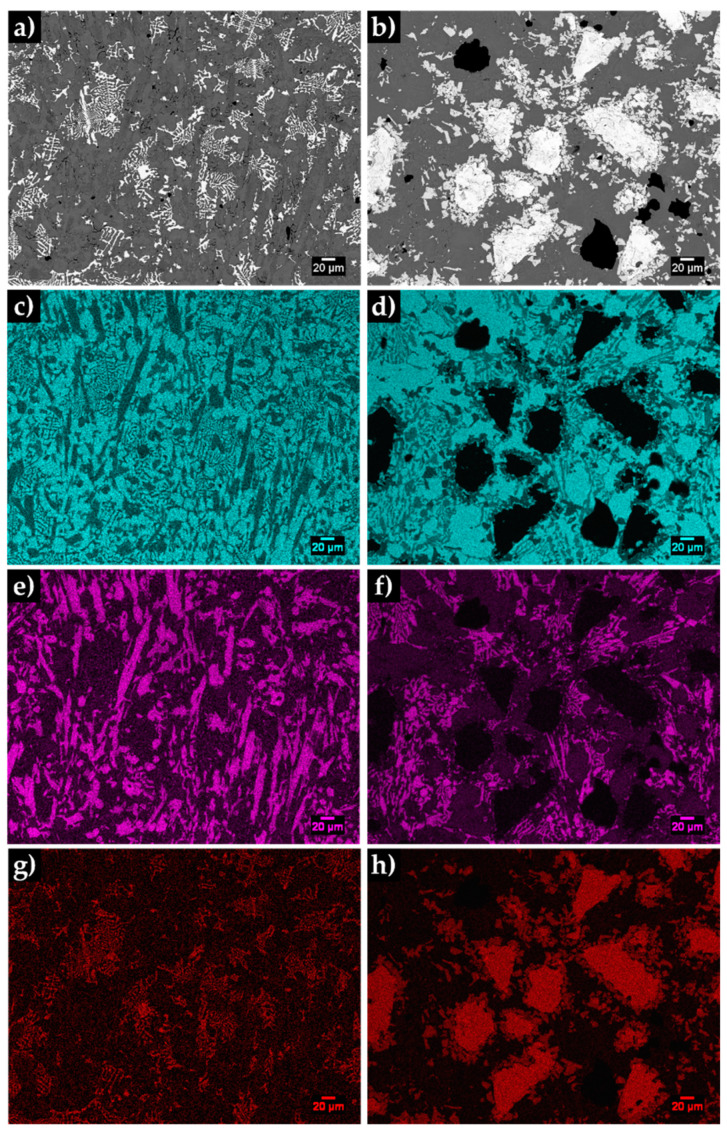
SEM-BSE images of the microstructure of the reaction layer (**a**) and composite zone (**b**). EDS elemental mapping (**c-h**) of Fe (blue), Cr (pink) and W (red).

The composite matrix is shown in Figure 14, the microstructure is characterized by a homogeneous and random distribution of white particles with a polygonal shape and larger dimension (typically between 50 and 200 µm) and small light gray particles (less than 10 µm) with an irregular shape. Elemental mapping analysis (Figure 15) confirmed that both larger and smaller particles are rich in W (in red), suggesting that they are a type of W carbide. The small particles also contain Cr (in pink) and Fe (in blue) in their composition. Note that the dark grey particles of the reaction layer are rich in Cr. The EDS results of the matrix were found to be similar to that of the base metal, with zones rich in Fe and others rich in Cr corresponding to Cr carbides.

**Figure 16:**
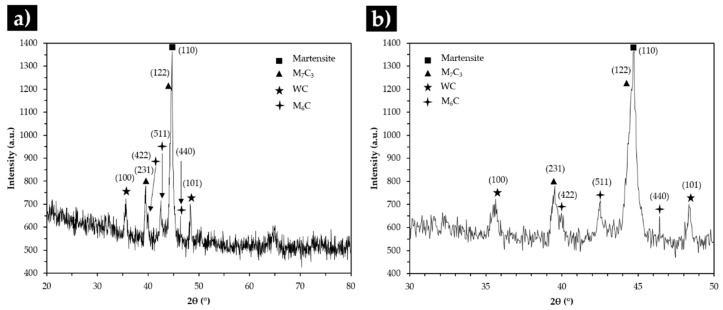
XRD patterns of the composite zone in the 2θ range of 20°–80° (**a**) and 30°–50° (**b**).

**Figure 17:**
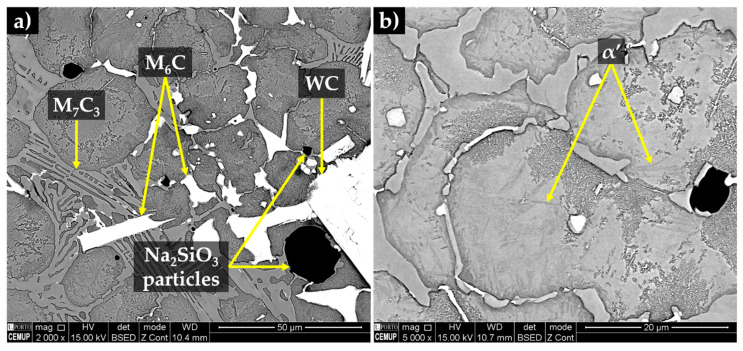
SEM-BSE images of the microstructure of the composite zone; (**a**) a network of eutectic constituent ((Fe,Cr)_7_C_3_ and (Fe,W,Cr)_6_C + α’), (Fe,W,Cr)_6_C next to the WC carbides and globules of Na_2_SiO_3_ and (**b**) martensite (α’).

**Figure 18:**
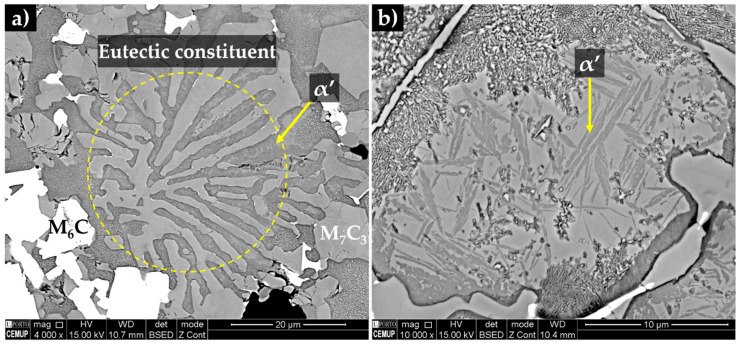
SEM-BSE images of the microstructure of the composite zone, (**a**) a detailed of a eutectic constituent (Fe,Cr)_7_C_3_ and (Fe,W,Cr)_6_C + α’) and (**b**) the martensite phase.

The XRD analysis (see Figure 16) permitted to identify the presence of WC, (Fe,Cr)_7_C_3_ and (Fe,W,Cr)_6_C phases in the reaction layer and composite zone. By combining with EDS analysis, one can deduce that the (Fe,W,Cr)_6_C particles have precipitated from the molten metal that surrounded and partially dissolved the WC carbides. Moreover, the precipitation of (Fe,W,Cr)_6_C carbides along with the reduction of Cr and C in the molten metal, leads to the hypoeutectic solidification structure, consisting of primary austenite and eutectic constituent with a network morphology that can be observed in Figure 17. Based on XRD and EDS analysis, the eutectic constituent is composed by two types of carbides: (Fe,Cr)_7_C_3_ and (Fe,W,Cr)_6_C and γ that transforms into martensite during the subsequent cooling (see Figure 17b and Figure 18). In Figure 17, it is also possible to observe the presence of black globules, that correspond to the sodium silicate particles, suggesting that the binder used has remelted and crystalized during the casting process due to the heat released by the liquid metal [25].

**Figure 19:**
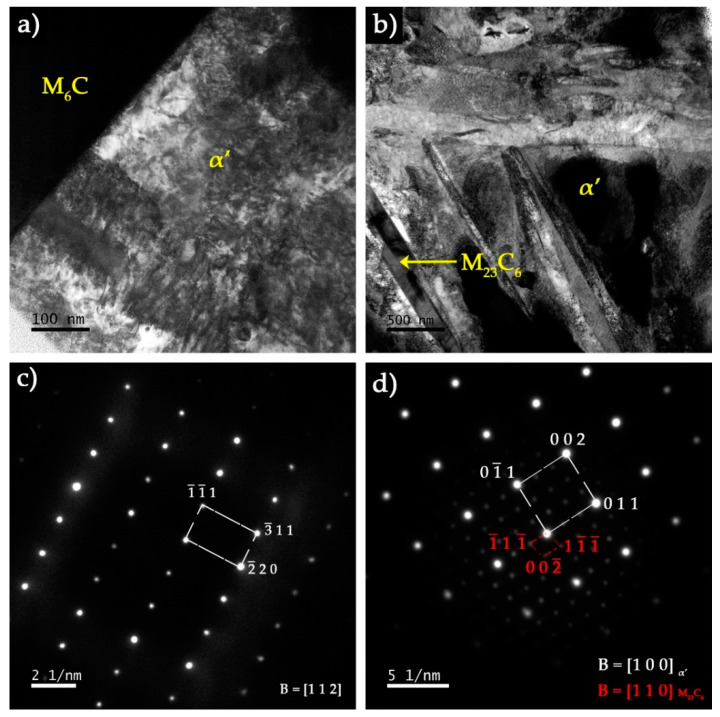
Dark-field TEM image of the composite showing (**a**) an interface between (Fe,W,Cr)_6_C carbide and α’ phase and (**b**) an M_23_C_6_ carbide embedded in α’. The identification of phases was performed by SAED analysis with (**c**) [112] zone axis of (Fe,W,Cr)_6_C and (**d**) [100] zone axis of α’ and [110] zone axis of M_23_C_6_.

TEM/SAED analysis was performed to further characterize the microstructural features of the constituents of the composite zone. Figure 19a shows the martensite matrix next to a large (Fe,W,Cr)_6_C particle. The contrast level of the martensite varies from light grey to black gray due to the high density of twins in the plate structure. In addition, thin plate-like M_23_C_6_ carbides that may have precipitated from martensite were observed in some localized regions (see Figure 19b).

## 4. Conclusions

High-Cr WCI specimens locally reinforced with WC–metal matrix composites were successfully produced via an ex-situ technique, using powder mixtures of WC and Fe and Na_2_SiO_3_ as a binder. The nonexistence of voids and porosities in the composite zone suggests a good infiltration of the molten metal into the inserted green compact. Moreover, the presence of a defect-free reaction zone, with about 200 µm of thickness, between the base metal and the composite zones should guarantee a strong bonding between these two regions. The microstructure of the composite zone shows a matrix with WC and (Fe,W,Cr)_6_C. The (Fe,W,Cr)_6_C precipitation was accompanied by a reduction of Cr in the base metal, leading to a hypoeutectic solidification. The final microstructure of the matrix is martensite and a eutectic constituent, composed of martensite and two types of carbides: (Fe,Cr)_7_C_3_ and (Fe,W,Cr)_6_C.

## Figures and Tables

**Table 1:** Nominal chemical composition of the high-Cr white cast iron (WCI) (wt.%).

C	Si	Mn	Cr	Ni	Fe
3.07	0.78	0.65	26.02	0.20	Balance

## References

[1] Doğan Ö., Hawk J., Laird G. (1997). Solidification structure and abrasion resistance of high chromium white irons. Metall. Mater. Trans. A.

[2] Tabrett C.P., Sare I.R., Ghomashchi M.R. (1996). Microstructure-property relationships in high chromium white iron alloys. Int. Mater. Rev..

[3] Heino V., Kallio M., Valtonen K., Kuokkala V.-T. (2017). The role of microstructure in high stress abrasion of white cast irons. Wear.

[4] Karantzalis E., Lekatou A., Mavros H. (2009). Microstructure and properties of high chromium cast irons: Effect of heat treatments and alloying additions. Int. J. Cast Met. Res..

[5] Davis J.R., Committee A.S.M.I.H., Davis J.R. (1996). Metallurgy and Properties of High-Alloy White Irons. ASM Specialty Handbook: Cast Irons.

[6] Wiengmoon A., Pearce J., Chairuangsri T. (2011). Relationship between microstructure, hardness and corrosion resistance in 20 wt.%Cr, 27 wt.%Cr and 36 wt.%Cr high chromium cast irons. Mater. Chem. Phys..

[7] Carpenter S., Carpenter D., Pearce J. (2004). XRD and electron microscope study of an as-cast 26.6% chromium white iron microstructure. Mater. Chem. Phys..

[8] Bedolla-Jacuinde A., Arias L., Hernández B. (2003). Kinetics of secondary carbides precipitation in a high-chromium white iron. J. Mater. Eng. Perform..

[9] Tang S., Gao Y., Li Y. (2014). Recent developments in fabrication of ceramic particle reinforced iron matrix wear resistant surface composite using infiltration casting technology. Ironmak. Steelmak..

[10] Hou S., Bao C., Zhang Z., Bai Y. (2013). Microstructure and Wear Behavior of High-Cr WCI Matrix Surface Composite Reinforced with Cemented Carbide Rods. J. Mater. Eng. Perform..

[11] Kambakas K., Tsakiropoulos P. (2006). Sedimentation casting of wear resistant metal matrix composites. Mater. Sci. Eng. A.

[12] Zhang P., Zeng S., Zhang Z., Li W. (2013). Microstructure and hardness of WC-Co particle reinforced iron matrix surface composite. China Foundry.

[13] Li Z., Jiang Y., Zhou R., Chen Z., Shan Q., Tan J. (2014). Effect of Cr addition on the microstructure and abrasive wear resistance of WC-reinforced iron matrix surface composites. J. Mater. Res..

[14] Leibholz R., Robert M.H., Leibholz H., Bayraktar E. (2017). Development of functionally graded nodular cast iron reinforced with recycled WC particles. Mechanics of Composite and Multi-functional Materials.

[15] Kambakas K., Tsakiropoulos P. (2005). Solidification of high-Cr white cast iron–WC particle reinforced composites. Mater. Sci. Eng. A.

[16] Niu L., Xu Y., Wang X. (2010). Fabrication of WC/Fe composite coating by centrifugal casting plus in-situ synthesis techniques. Surf. Coat. Technol..

[17] Zhang Z., Chen Y., Zuo L., Zhang Y., Qi Y., Gao K., Liu H., Wang X. (2018). In situ synthesis WC reinforced iron surface composite produced by spark plasma sintering and casting. Mater. Lett..

[18] Pierson H.O. (1996). Carbides of Group VI: Chromium, Molybdenum, and Tungsten Carbides. Handbook of Refractory Carbides and Nitrides: Properties, Characteristics, Processing and Applications.

[19] (2019). CES EduPack—Database: Level 3, 19.1.0.

[20] Zhou R., Jiang Y., Lu D. (2003). The effect of volume fraction of WC particles on erosion resistance of WC reinforced iron matrix surface composites. Wear.

[21] Cuevas A.C., Becerril E.B., Martínez M.S., Ruiz J.L. (2018). Fabrication Processes for Metal Matrix Composites. Metal Matrix Composites: Wetting and Infiltration.

[22] Manu K.S., Raag L.A., Rajan T., Gupta M., Pai B. (2016). Liquid Metal Infiltration Processing of Metallic Composites: A Critical Review. Metall Mater Trans B.

[23] Chawla N., Chawla K.K. (2013). Processing. Metal Matrix Composites.

[24] Cao G.J., Guo E.J., Feng Y.C., Wang L.P. (2012). Abrasion Behavior of WC Reinforced Cast Iron Surface Composite Fabricated by Cast-Infiltration Method. Advanced Materials Research.

[25] Aso S., Goto S., Komatsu Y., Ike H., Shimizu K. (2003). Composite reinforcement of the surface of cast iron by WC powder inserts. Int. J. Cast Met. Res..

[26] Leibholz R., Leibholz H., Bayraktar E., Robert M.H. (2019). Investigation on Microstructure and Interfaces in Graded FE50007/WC Composites Produced by Casting. Mechanics of Composite, Hybrid and Multifunctional Materials.

[27] Zhang G.-S., Xing J.-D., Gao Y.-M. (2006). Impact wear resistance of WC/Hadfield steel composite and its interfacial characteristics. Wear.

[28] Zhang G.S., Gao Y.M., Xing J.D., Wei S.Z., Zhang X.L. (2007). Interfacial Characteristics and Wear Resistance of WCp/White-Cast-Iron Composites. Adv. Mater. Res..

[29] Song Y., Wang H. (2012). High speed sliding wear behavior of recycled WCP-reinforced ferrous matrix composites fabricated by centrifugal cast. Wear.

[30] Chumanov I., Anikeev A., Chumanov V. (2015). Fabrication of functionally graded materials by introducing wolframium carbide dispersed particles during centrifugal casting and examination of FGM’s structure. Procedia Eng..

[31] Niu L., Hojamberdiev M., Xu Y. (2010). Preparation of in situ-formed WC/Fe composite on gray cast iron substrate by a centrifugal casting process. J. Mater. Process. Technol..

[32] Zheng K., Gao Y., Tang S., Li Y., Ma S., Yi D., Zhang Z. (2014). Interface Structure and Wear Behavior of Cr26 Ferrous Matrix Surface Composites Reinforced with CTCp. Tribol. Lett..

[33] Li Y., Gao Y. (2010). Three-body abrasive wear behavior of CC/high-Cr WCI composite and its interfacial characteristics. Wear.

[34] Shan Q., Li Z., Jiang Y., Zhou R., Sui Y. (2013). Effect of Ni addition on microstructure of matrix in casting tungsten carbide particle reinforced composite. J. Mater. Sci. Technol..

[35] Li Z.L., Chen Z.H., Jiang Y.H., Zhou R., Shan Q., Song Q.L. (2012). Influence of Addition of Tungsten-iron powder on Microstructure of WC/steel Composite Coatings. Advanced Materials Research.

[36] Li Z., Jiang Y., Zhou R., Gao F., Shan Q., Tan J. (2014). Thermal fatigue mechanism of WC particles reinforced steel substrate surface composite at different thermal shock temperatures. J. Alloys Compd..

[37] Li Z., Jiang Y., Zhou R., Lu D., Zhou R. (2007). Dry three-body abrasive wear behavior of WC reinforced iron matrix surface composites produced by V-EPC infiltration casting process. Wear.

[38] Huang R.Q., Li Z.L., Jiang Y.H., Zhou R., Gao F. (2012). Thermal Shock Cracks Initiation and Propagation of WCp/Steel Substrate Surface Composite at 500 °C. Appl. Mech. Mater..

[39] Sui Y., Han L., Jiang Y., Li Z., Shan Q. (2018). Effects of Ni60WC25 powder content on the microstructure and wear properties of WCp reinforced surface metal matrix composites. Trans. Indian Inst. Met..

[40] Moreira A.B., Sousa R.O., Lacerda P., Ribeiro L.M.M., Pinto A.M.P., Vieira M.F. (2020). Microstructural Characterization of TiC–White Cast-Iron Composites Fabricated by In Situ Technique. Materials.

[41] (2010). Standard Specification for Abrasion-Resistant Cast Irons. A532/A532M—10.

[42] Laird G., Gundlach R., Rohrig K. (2000). Abrasion-Resistant Cast Iron Handbook.

[43] Suwas S., Ray R.K., Derby B. (2014). Crystallographic Texture of Materials.

[44] Liu T., Ping D., Ohmura T., Ohnuma M. (2018). Electron diffraction analysis of quenched Fe–C martensite. J. Mater. Sci..

[45] Ping D., Guo S., Imura M., Liu X., Ohmura T., Ohnuma M., Lu X., Abe T., Onodera H. (2018). Lath formation mechanisms and twinning as lath martensite substructures in an ultra low-carbon iron alloy. Sci. Rep..

[46] Zhang P., Chen Y., Xiao W., Ping D., Zhao X. (2016). Twin structure of the lath martensite in low carbon steel. Prog. Nat. Sci..

[47] Ping D., Ohnuma M. (2018). ω-Fe particle size and distribution in high-nitrogen martensitic steels. J. Mater. Sci..

